# The effect of varying intensities of lower limb eccentric muscle contractions on left ventricular function

**DOI:** 10.1007/s00421-019-04298-0

**Published:** 2020-01-16

**Authors:** Luke A. Howlett, Kyle O’Sullivan, Nicholas Sculthorpe, Joanna Richards

**Affiliations:** 1 Institute of Sport Science and Physical Activity Research (ISPAR), University of Bedfordshire, Bedford, MK41 9EA UK; 2 Institute for Clinical Exercise and Health Science, University of the West of Scotland, Hamilton, ML3 0JB UK; 3 School of Biomedical Sciences, Faculty of Biological Sciences, University of Leeds, Leeds, LS2 9JT UK

**Keywords:** Eccentric exercise, Left ventricle, Cardiovascular, Wall stress, Longitudinal strain

## Abstract

**Purpose:**

The effect of eccentric (ECC) resistance exercise (RE) on myocardial mechanics is currently unknown.

**Method:**

This study investigated ECC RE at varying intensities on left ventricular (LV) function using LV strain (ε), wall stress and haemodynamic parameters. Twenty-four healthy male volunteers completed ECC leg extensions at 20%, 50% and 80% of their ECC maximal voluntary contraction (MVC), whilst receiving echocardiograms. Global longitudinal ɛ, strain rate (SR), longitudinal tissue velocity, heart rate (HR), blood pressure (BP), mean arterial pressure (MAP), LV wall stress and rate pressure product (RPP) were assessed at baseline and during exercise.

**Results:**

Left ventricular global ɛ, systolic SR and wall stress remained unchanged throughout. Systolic blood pressure (sBP), MAP and RPP increased at 80% and 50% intensities compared to rest (*P* < 0.01). Eccentric RE increased HR and peak late diastolic SR at all intensities compared to rest (*P* < 0.02).

**Conclusion:**

The findings suggest acute ECC RE may not alter main parameters of LV function, supporting future potential for wider clinical use. However, future studies must investigate the impact of multiple repetitions and training on LV function.

## Introduction

In clinical practice, the current trans-thoracic echocardiogram evaluates LV cavity size and wall thickness to assess structural components; whilst valve health, inflow velocity and ejection fraction are investigated to understand function (Wharton et al. 2015). However, technological advances have enabled the validation of new techniques such as speckle tracking imaging (STI) (Yamada et al. 2014). Speckle tracking imaging allows investigation of LV strain (ε) which is defined as the deformation of the LV through contraction and relaxation of cardiomyocytes (Farsalinos et al. 2015; Kalam et al. 2014). Research during the last decade has established longitudinal ε as an effective indicator of LV systolic function (Krishnasamy et al. 2015). It has also been considered a superior predictor of cardiovascular mortality when compared to ejection fraction (Kalam et al. 2014; Krishnasamy et al. 2015; Farsalinos et al. 2015); with suggestions for potential clinical use (Smiseth et al. 2016; Fine et al. 2014; Farsalinos et al. 2015). In addition, global longitudinal ε has been suggested to have prognostic value in determining subclinical LV impairments (Potter and Marwick 2018). With mounting evidence supporting the clinical diagnostic potential of resting LV global longitudinal ε, a logical progression is to determine if exercise stress could prove useful in uncovering LV function decrements. Exercise tests are commonly used to aid diagnoses as exercise stress may uncover systolic or diastolic dysfunction not evident at rest (Hill and Timmis 2002). Therefore, it is plausible that myocardial ɛ evaluation during exercise may also be more discriminating between normal and pathological cardiac function (Kalam et al. 2014; Krishnasamy et al. 2015; Farsalinos et al. 2015). However, whilst normal resting values have been documented in previous literature (− 14 to − 23%) (Yingchoncharoen et al. 2013; Levy et al. 2016; Kocabay et al. 2014; Moreira et al. 2017), normal values during exercise stress have not been established, and there has been little consideration of the varied exercise modalities commonly performed recreationally such as resistance exercise (RE) as well as aerobic exercise. Understanding normal ε values during these activities may also help in the development of therapeutic strategies as well as in diagnostics.

The incorporation of RE alongside aerobic exercise in exercise programs of clinical populations has become more common given the benefits of RE and in recent years the benefits of RE types other than concentric (CON) RE, such as isometric and eccentric (ECC) have been investigated. Isometric exercise can prevent or delay significant muscle mass and strength reductions (Hettinger 2017) and improve blood pressure control (Carlson et al. 2014). However, studies evaluating ECC RE modalities suggest this exercise type may be superior, facilitating beneficial increments in musculoskeletal, metabolic and cardiovascular function (Isner-Horobeti et al. 2013). Research in this area suggests ECC RE markedly improves muscle mass, muscle strength (Roig et al. 2009; Farthing Chilibeck 2003), insulin sensitivity and physical fitness (Chen et al. 2017) with reduced associated cardiovascular and metabolic stress compared to matched CON RE protocols (Overend et al. 2000; Vallejo et al. 2006). Thus, the potential use of ECC RE in populations with cardiovascular conditions and/ or reduced exercise capacities has been postulated (Mitchell et al. 2017). Although precise mechanisms are currently unknown, physiological differences between ECC and CON RE have been reported to result from superior motor unit recruitment, force production, differential cell signalling (mitogen-activated protein kinase: MAPK), and enhanced protein synthesis following ECC RE (Mitchell et al. 2017).

Previous work has investigated different types of RE, for example Stefani et al. (2008) reported greater apical longitudinal strain during a 3-min moderate intensity isometric handgrip exercise (30% maximal handgrip strength) in trained athletes compared to untrained individuals (Stefani et al. 2008). Contrasting, studies have reported acute declines in LV contractility and LV systolic function during lower limb bilateral isometric (10 s maximal leg raise) (Alegret et al. 2015) and bilateral leg press RE at higher intensities (30–60% 1RM) (Stöhr et al. 2017). One explanation for the different findings is the differences in exercise intensities used.

Given these disparities in findings more studies are necessary to better understand the longitudinal ε dynamics of the LV during ECC type RE. Further investigation will benefit understanding of this exercise type and may aid development of more advanced therapeutic and diagnostic strategies, alongside assisting in the generation of exercise reference values. Therefore, this study will investigate the effect of exercise intensity during lower limb ECC RE on LV function. This study will aim to evaluate LV global ε, SR and wall stress during an ECC leg extension at 20%, 50%, and 80% of the ECC maximal voluntary contraction (MVC). It is hypothesised that there will be no significant difference in Global longitudinal ε during ECC RE independent of intensity compared to baseline.

## Methodology

### Participants

Twenty-four recreationally active healthy males volunteered to participate in this study. General participant characteristics displayed in Table. 1. All participants were provided with study information sheets prior to completing informed consent, physical activity readiness questionnaires (PAR-Q) and health screening questionnaires, on arrival at the University of Bedfordshire sport science laboratories. Participants were excluded from the study if they had any cardiovascular, pulmonary or metabolic conditions, epilepsy, musculoskeletal injuries, recent surgery or chest pain upon exercise. This study was approved by the University of Bedfordshire ethics committee which conforms to the Declaration of Helsinki.

**Table 1:** Participant physical characteristics

	Mean	Standard deviation
Age (year)	23	4
Height (cm)	179	6
Body mass (kg)	83.1	18.8
Body fat (%)	23.1	10.4
BMI (kg.cm^2^)	25.9	5.3

### Experimental design

This was a repeated-measures research design. Participants visited the laboratory in light training appropriate clothing, having refrained from alcohol, strenuous physical activity and caffeine for 24 h prior. Participants completed two visits, one preliminary and one main trial session separated by 5–7 days. During the preliminary visit, participants performed an ECC knee extension maximal voluntary contraction (MVC) using an isokinetic dynamometer and received body fat (BF) percentage measurements via air-displacement plethysmography. During the main trial visit, participants rested for a period of 5 min in the supine position and received a transthoracic echocardiogram at baseline and during ECC knee extensions at 20%, 50%, and 80% of the pre-determined ECC MVC. During each echocardiogram, heart rate (HR) and blood pressure (BP) were measured.

### Maximal voluntary contraction

Participants’ height (cm) was recorded with shoes removed using a wall-mounted stadiometer (Harpenden, HAR- 98.602, Holtain). Resting BP measurements were acquired using an aneroid non-mercury sphygmomanometer (Riester, Big Ben, Medisave) following standard guidelines by the American Heart Association (AHA) and the British Hypertension Society (BHS) (Pickering et al. 2005; Williams et al. 2004). All ECC MVC manoeuvres were performed from the supine position on an isokinetic dynamometer (Chattecx Corporation, Kin Com 125E Plus, Chattanooga) after completion of a 5-min cycling warm up at 70 W using a cycle ergometer (Monark, 824E, Cranlea). The ankle brace and the axis of the isokinetic dynamometer were aligned with the lateral malleolus and lateral tibial condyle, respectively, for each participant. The performance of the ECC MVC required the participant to produce maximal force against the axis as it moved from 150–160° to 90° at a rate of 10°s^−1^. Three ECC MVC manoeuvres were performed and the highest peak force (N) was recorded (mean ECC MVC: 1141 ± 342 N). Two-minute rests separated each attempt. The session was terminated at the completion of a 3-min cycling cool down at 70 W.

### Air-displacement plethysmography

Participants wore minimal clothing, such as swim wear and cap for the duration of the procedure. Participants were required to stand on the calibration scales to attain body mass (kg) and sit inside the Bod Pod (Bod Pod; 2000A; Cranlea) for 3 min to obtain BF percentage. This was repeated 1 further time. Body fat was deemed reproducible only when body volume differences < 150 ml (Vescovi et al. 2001). If body volume was > 150 ml between tests, further tests were performed. No specific fasting was required prior assessment (Orlandi et al. 2013).

### Eccentric leg extension

Warm up and equipment set up was the same as for the ECC MVC during the preliminary visit. Participants were required to perform ECC leg extensions from the supine position upon the isokinetic dynamometer eliciting forces against the axis equivalent to 20% (mean: 228 ± 68 N), 50% (mean: 571 ± 171 N) and 80% (mean: 913 ± 274 N) (Goto et al. 2009; Isner-Horobeti et al. 2013; Farthing Chilibeck 2003; Colberg et al. 2010) of the pre-determined ECC MVC using the attached visual feedback screen. Each manoeuvre, at all intensities, were repeated a minimum of two times (one for each image capture). Axis speed and range of movement were fixed at 10°.s^-1^ and 150–160° to 90°, respectively. Participants received a countdown prior to initiation of all manoeuvres to prevent premature exertion.

### Rest and exercise echocardiography

**Fig. 1:**
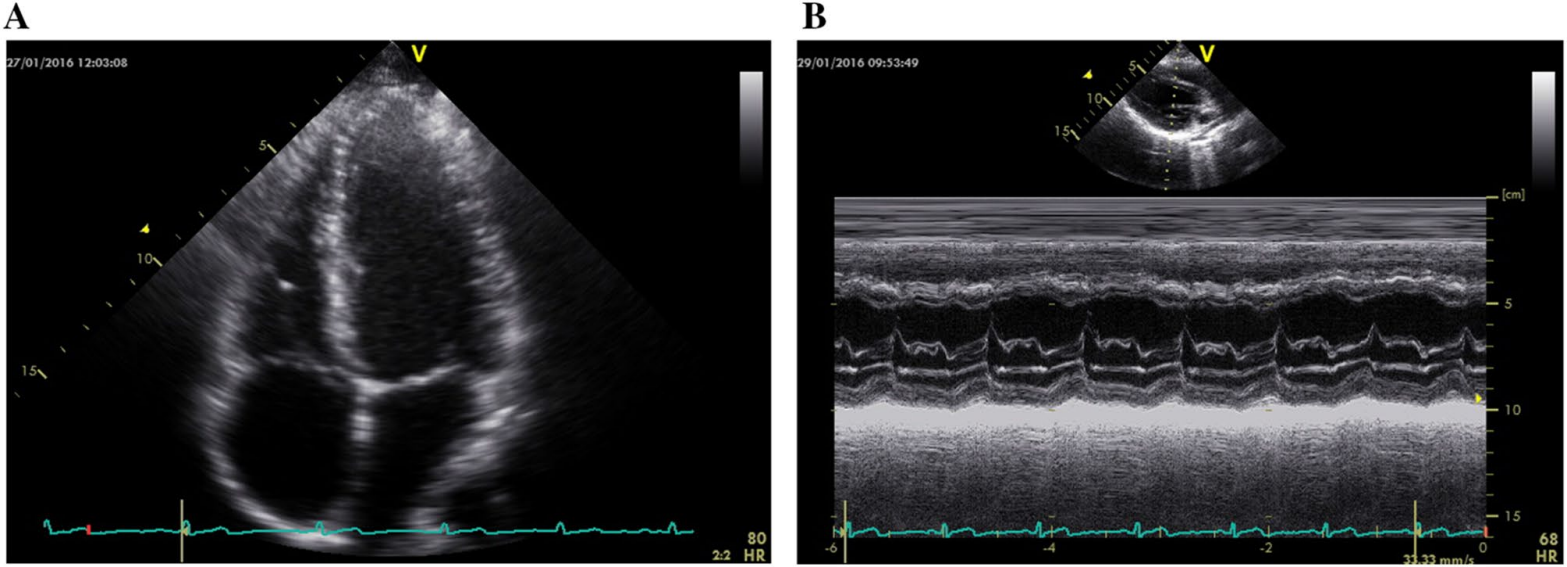
**a** Experimental frame extracted from a captured cineloop of the apical 4 chamber view. **b** Experimental image capture of the 4-chamber parasternal long axis view observed during M-mode recording

**Fig. 2:**
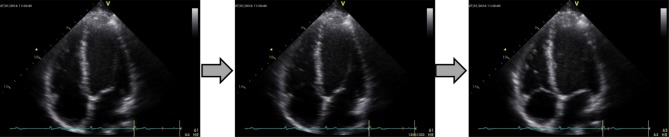
Schematic displaying frames extracted from a cineloop recording, representing LV deformation during a cardiac cycle at rest

All cardiac investigations were performed with the participant in the supine position in a partial left lateral decubitus position upon an isokinetic dynamometer, using an echocardiograph system (1.5–4 MHz. Phased Array transducer, GE Medical, Vivid 7). A 3 lead ECG (GE Medical, Vivid 7) was used continuously to monitor HR and track cardiac cycles for offline ε analysis. Heart rate was recorded as the peak HR occurring during the ECC contraction. Images were captured (Fig. 1) at a frame rate just below 90 frames per second via Motion mode (M-mode) from the tips of the mitral valve in the 4-chamber parasternal long axis view, whilst cineloops were captured of the apical 4 chamber view (Fig. 2) (Wharton et al. 2015). Image quality of cineloops for ε analysis were measured; Seventy three percent of all cineloops were of satisfactory to excellent quality. End-diastolic LV diameter (LVDd), end-diastolic interventricular septal thickness (IVSd) and end-diastolic posterior wall thickness (PWd) were measured offline using M-mode images. Left ventricular wall stress, mean arterial pressure (MAP) and rate of pressure product (RPP) were calculated in accordance with ASE criteria.

**Fig. 3:**
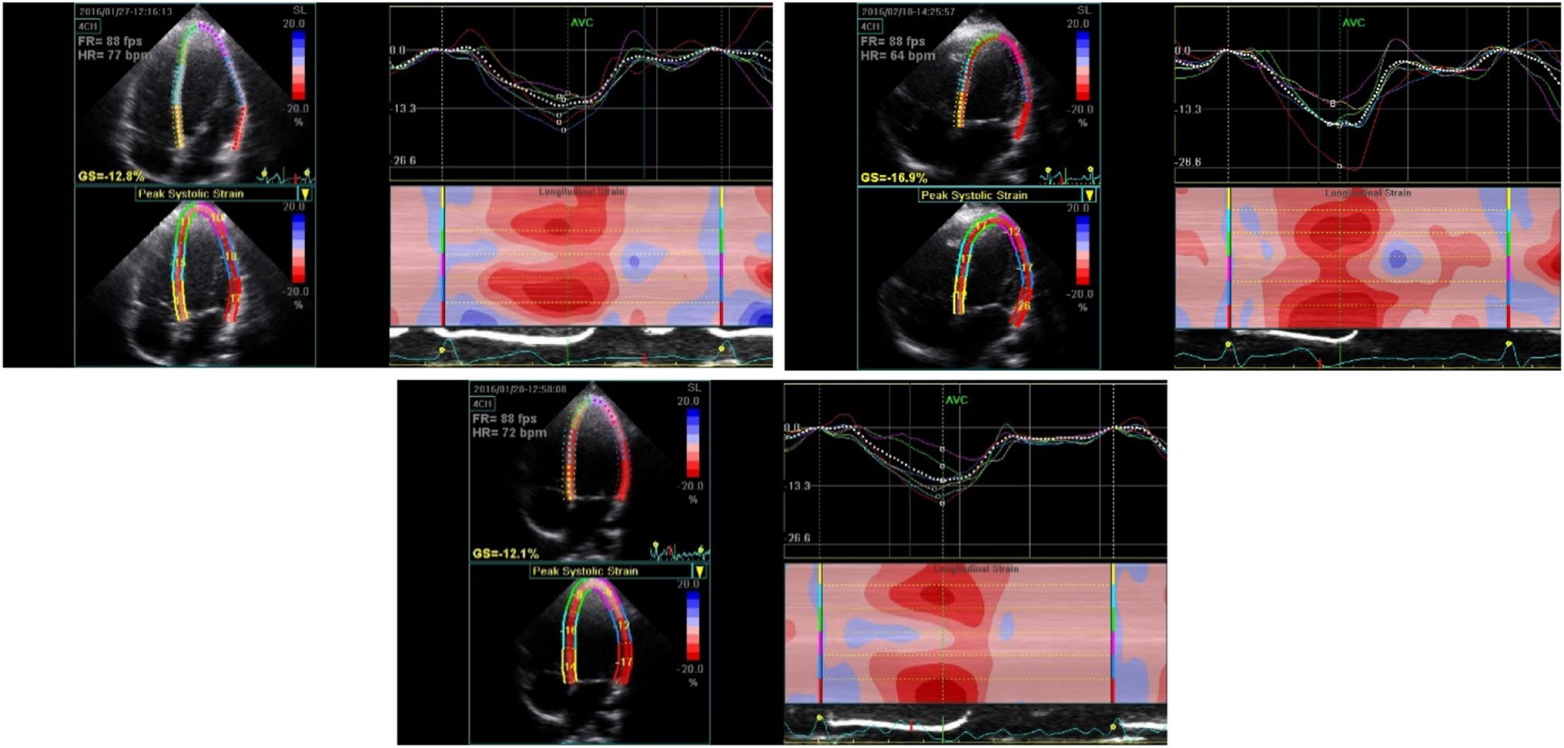
Experimental traces measuring global longitudinal ε offline using STI techniques

All ε analyses weres performed through semi-automated STI (Fig. 3) (Dandel and Hetzer 2009; Brown et al. 2009; Stewart et al. 2016), offline using EchoPac software (EchoPac software, GE Health Care, Version 113, GE Health Care). Strain-related variables were recorded as an average over 3 consecutive cardiac cycles for each individual at rest. During all exercise intensities strain analysis was recorded as an average over 3 consecutive cardiac cycles as close to the cessation of the ECC contraction as possible. The maximum percentage deformation of the LV was recorded as the peak global longitudinal ε. The maximum rate of LV deformation during systole, early diastole and late diastole were recorded as peak systolic strain rate (SR), peak early diastolic SR and peak late diastolic SR, respectively. The maximum velocity of LV deformation during early and late diastole were recorded as peak longitudinal early diastolic velocity and peak longitudinal late diastolic velocity, respectively. Reliability of longitudinal ε using STI was ensured with the coefficient of variation residing at 7.9% and 13.9% for rest and exercise measurements, respectively. Baseline echocardiography images were recorded after a minimum of 5 min rest. Exercise echocardiography images and cineloops were acquired during eccentric leg extensions at 20%, 50%, and 80% of the identified ECC MVC and up to 5 cardiac cycles after cessation of each repetition. Blood pressure was measured manually at rest. Exercising blood pressure measurements took place during ECC contractions and up to 10 s post.

### Statistical analysis

A one-way repeated-measures analysis of variance (ANOVA) compared: HR, sBP, diastolic blood pressure (dBP), LV global longitudinal ε, LV SR, LV longitudinal tissue velocity, LV wall stress, MAP and RPP in all subjects (*N* = 24) between rest and 20%, 50% and 80% exercise intensities in this repeated-measures test design. All relevant assumptions were met. Bonferroni post-hoc analysis identified where differences were situated. Global longitudinal ε coefficient of variation (CV) was calculated via pairwise analysis of reliability data following Hopkins (2015) model and reflected low variability (Decloedt et al. 2011). All statistical analyses were performed using IBM statistics 22 software (SPSS Inc., Chicago, IL). GraphPad Prism 8 was used to create graphs. All data presented as mean ± standard deviation (SD). Statistical significance was considered at *P* < 0.05.

## Results

**Fig. 4:**
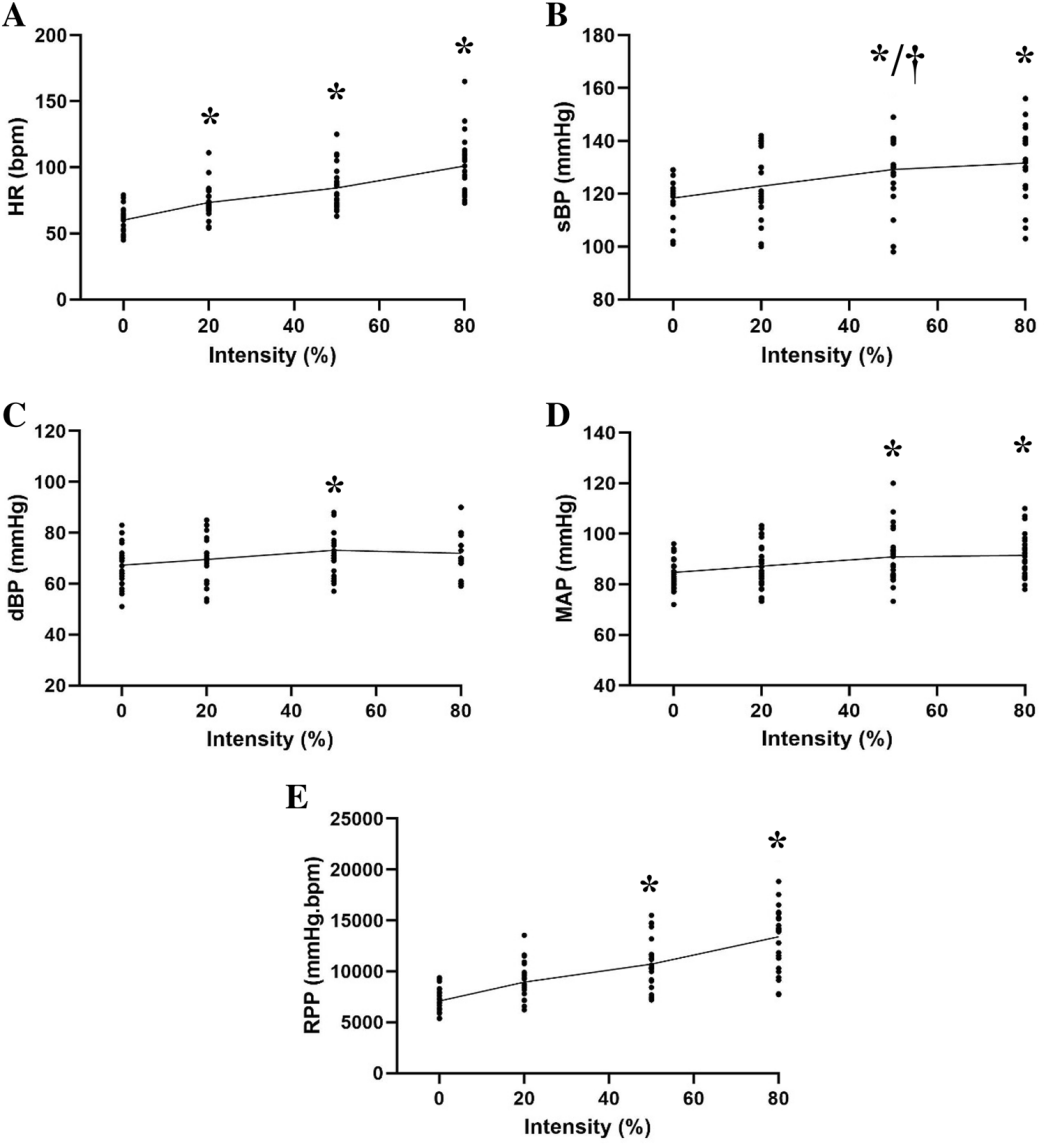
Haemodynamic profiles, with mean trendlines, during different intensities of ECC RE. **a** Heart rate (HR). **b** Systolic blood pressure (sBP). **c** Diastolic blood pressure (dBP). **d** Mean arterial pressure (MAP). **e** Rate pressure product (RPP). * Denotes significant change compared to rest (*p* < 0.01). † denotes significant change compared to 20% intensity (*p* < 0.01)

Eccentric RE stimulated a significant increase in HR (*P* < 0.001; Fig. 4) at all intensities compared to rest (rest: 60 ± 9 vs 20%: 73 ± 13 vs 50%: 84 ± 16 vs 80%: 101 ± 22 bpm). Significant increases in sBP were also identified at 80% and 50% intensity ECC RE (*P* < 0.001) in comparison to rest (80%: 132 ± 13 vs 50%: 129 ± 14 vs rest: 118 ± 8 mmHg). Furthermore, there were significant increments (P = 0.004) in sBP between 20 and 50% intensity exercise (20%: 123 ± 12 vs 50%: 129 ± 14 mmHg). Moreover, a significant increase in dBP (*P* = 0.008) was found at 50% intensity ECC RE compared to rest (50%: 73 ± 11 vs rest: 67 ± 8 mmHg). In addition, both MAP and RPP displayed significant increments (*P* < 0.005; *P* < 0.005) at 50% and 80% intensities compared to rest (MAP, rest: 85 ± 6 vs 50%: 91 ± 11 vs 80%: 91 ± 9 mmHg; RPP, rest: 7091 ± 1165 vs 50%: 10,175 ± 2927 vs 80%: 13,405 ± 3440 mmHg bpm).

**Fig. 5:**
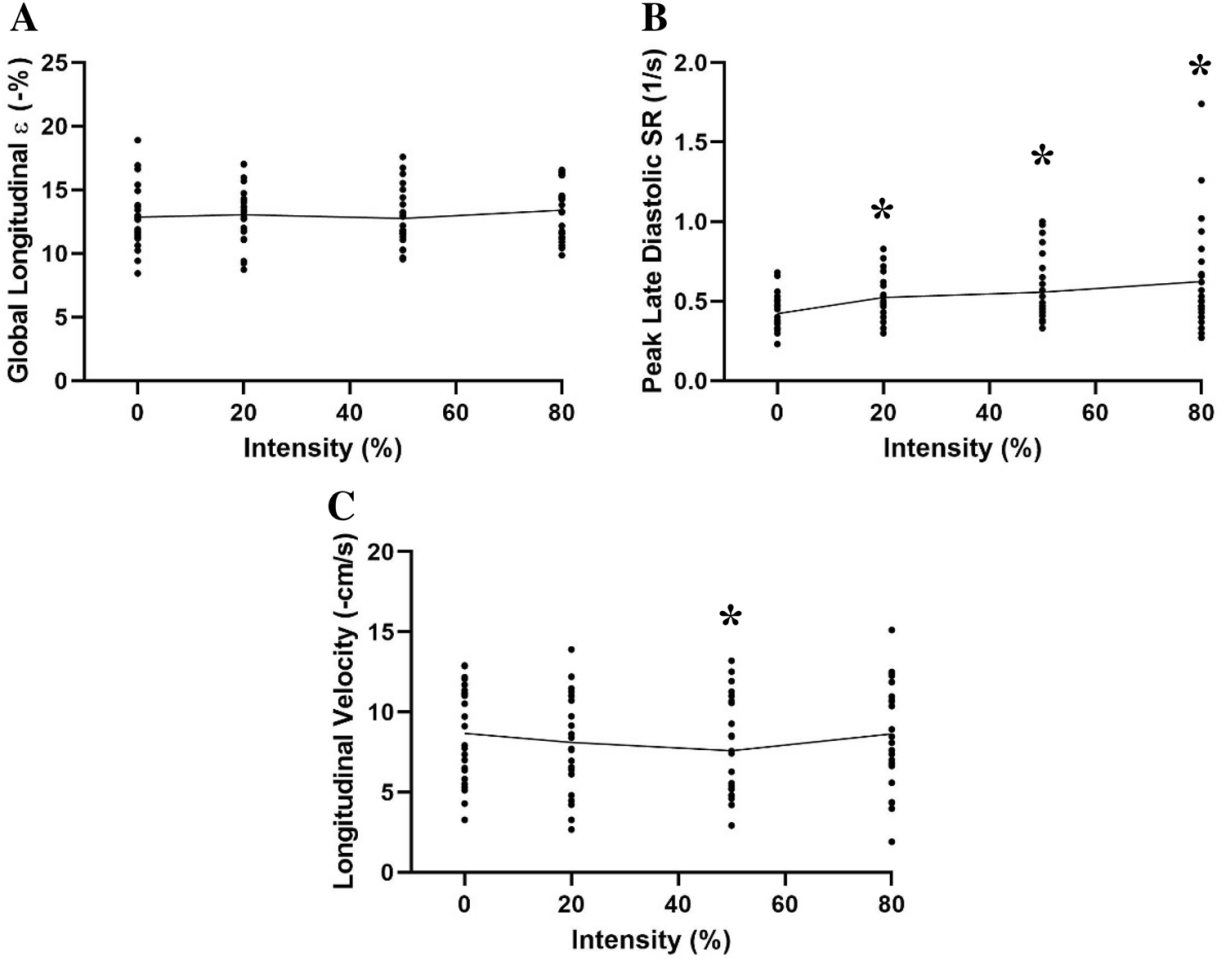
LV **a** global longitudinal strain (ε); **b** peak late diastolic strain rate (SR) and **c** peak early diastolic longitudinal velocity profiles with mean trendlines during different intensities of ECC RE. * Denotes significant change compared to rest (*p* < 0.05)

Progressive ECC RE did not significantly change (*P* = 0.245; Fig. 5) LV global longitudinal ε during any exercise intensity (rest: − 12.9 ± 2.4 vs 20%: − 13.0 ± 2.2 vs 50%: − 12.8 ± 2.2 vs 80%: − 13.4 ± 2.2%). Peak systolic SR (rest: − 0.86 ± 0.16 vs 20%: − 0.93 ± 0.18 vs 50%: − 0.86 ± 0.18 vs 80%: − 0.79 ± 0.51 s ^− 1^; *P* = 0.37) and peak early diastolic SR (rest: 1.27 ± 0.28 vs 20%: 1.25 ± 0.30 vs 50%: 1.19 ± 0.26 vs 80%: 1.29 ± 0.34 s^−1^; *P* = 0.32) were unchanged during ECC RE, whilst peak late diastolic SR increased significantly (*P* =  < 0.02) at all exercise intensities compared to rest (rest: 0.42 ± 0.11 vs 20%: 0.52 ± 0.15 vs 50%: 0.56 ± 0.21 vs 80%: 0.62 ± 0.33 s^−1^). No significant changes were observed in peak late diastolic myocardial velocity (rest: − 3.51 ± 1.63 vs 20%: − 3.69 ± 2.73 vs 50%: − 3.70 ± 1.80 vs 80%: − 4.39 ± 1.76 cm/s; *P* = 0.09). However peak early diastolic velocity became less negative at 50% exercise intensity compared to rest (50%: − 7.60 ± 2.97 vs rest: − 8.67 ± 2.92 cm/s; *P* = 0.04). Finally, LV wall stress (*P* = 0.856) remained unchanged during ECC RE (rest: 130.38 ± 43.29 vs 20%: 127.27 ± 44.04 vs 50%: 129.62 ± 41.59 vs 80%: 127.66 ± 35.16 dyn cm^−2^).

## Discussion

The aim of this study was to investigate the effect of different intensities of ECC RE on LV longitudinal ɛ. ECC RE elicited positive chronotropic and haemodynamic responses without concomitant alterations in global longitudinal ɛ and systolic SR. The results of this study may suggest large muscle group single-repetition ECC RE transiently upregulates haemodynamic markers and HR responses, without augmenting LV systolic function. The lack of significant alterations in LV global longitudinal ɛ during ECC RE independent of intensity may support existing research which suggests ECC exercise stimulates reduced cardiovascular stress compared to traditional CON RE (Overend et al. 2000; Vallejo et al. 2006). First, such findings may suggest LV systolic function is unaffected during a single ECC RE repetition, which contrasts existing research investigating myocardial function during other forms of exercise and interestingly, during other forms of RE (Stefani et al. 2008; Stewart et al. 2016). Second, it may also indicate that single repetitions of ECC RE up to 80% of the ECC MVC, might not influence the myocardial contractile response in the same manner as more traditional aerobic and RE modalities. Conversely, the lack of alterations observed in global longitudinal ε in response to ECC RE may be a result of the measurement of ε during the performance of one repetition rather than multiple repetitions or sets as commonly performed recreationally. Thus, despite the results of this study suggesting no change after one repetition, the impact of performing multiple repetitions may be entirely different. Existing research indicates that haemodynamic responses to RE are cumulative and increase with motor unit recruitment, intensity and volume, which may facilitate an amplified cardiac response (Mayo and Kravitz 1999). Although this study reported unchanged systolic LV function during brief ECC RE (Fig. 5), significant increments were yielded in peak late diastolic SR (all intensities). This indicates that the ECC RE impacts late diastolic function, likely resulting from the elevated haemodynamic load (Fig. 4). The haemodynamic changes coupled with significant increments in peak late diastolic SR during ECC RE compared to rest, may suggest that cardiac function is indeed undergoing exercise induced alteration. However, changes in longitudinal ε may have been prevented or delayed due to increased afterload associated with large muscle group RE contractions (Mayo and Kravitz 1999). This is supported by research which has found LV ε to be inversely related to increases in afterload (Burns et al. 2009; Rösner et al. 2008). Although during our study, LV ε did not reduce, it remained unchanged. This could be because during a single repetition of lower limb ECC RE, inotropic drive and afterload are matched, offsetting any alterations in global longitudinal ε. Therefore, it may be that brief ECC RE performance does incur a physiological load, yet the heart is able to cope due to its transient nature without improvements in longitudinal ε. This might support the suggestion of a cumulative effect whereby the continuation of effort may have instigated enhanced systolic function in the form of LV ε.

The available research investigating LV function during RE displays contradicting findings for instance, Stefani et al. (2008) reported improvements in apical longitudinal ɛ in elite footballers during a handgrip exercise at 30% MVC for 3 min; yet Weiner et al. (2012) discovered significant reductions in global longitudinal ɛ and longitudinal SR in normal healthy individuals during an experimental protocol involving performing a handgrip exercise for 3 min at 40% MVC. The study by Weiner et al. (2012) also reported important reductions in LV twist mechanics and apical rotations. Such findings are supported by a more recent study conducted by Stöhr et al. (2017) which observed decrements in LV circumferential ɛ and circumferential SR during double leg press exercise at 30% and 60% MVC compared to rest. The findings of this study provide some contrast; evident from unchanged LV global longitudinal ε and LV wall stress. The contrasting findings are most likely a result of differences in exercise protocol, alongside varied types of measured myocardial ɛ, exercise intensity, different muscle group recruitment and more importantly exercise type.

It may be typically hypothesised that greater motor unit recruitment through the use of larger muscle groups—leg extension in this case—may trigger increased ventricular contractile alterations; however, the length of contraction as required by the two previously mentioned studies is far greater (3 min) and the intensity is markedly lower than commonly used in recreationally trained populations (Stefani et al. 2008; Weiner et al. 2012). Therefore, it may be argued that such an exercise type may trigger similar cardiovascular responses to endurance type exercise as opposed to RE (Stewart et al. 2016). In addition, while conceptually ECC RE and static RE forms (such as isometric and CON) are similar, the expected differences in cardiovascular response make it difficult to draw comparisons, especially when such contrasts in effort and contractile durations are apparent.

The haemodynamic responses discovered in this experiment coincide somewhat with the general cardiovascular responses understood to occur during a bout of exercise, although to a lesser amplitude. For instance, RE traditionally stimulates acute increases in HR, BP and arterial resistance which subsequently increases MAP and RPP (Stӧhr et al. 2015), due to increased sympathetic nervous system activation, respiratory drive and blood demand alongside periodic arterial occlusion and local vasodilation as a result of intermittent muscular contraction, respectively (Overend et al. 2000). Such increases in BP are supported in existing literature (Stefani et al. 2008; Weiner et al. 2012; Beaumont et al. 2017). Yet the HR and BP response is less exaggerated in this study (< 102 bpm), which may be a result of the reduced cardiovascular response to the ECC leg extension evident from the lack of effect of LV longitudinal ε and lesser haemodynamic response relative to exercise intensity. Similarly, low BP values were reported during ECC squatting exercise (65% MVC) by Vallejo et al. (2006), although contradicting results exist (Overend et al. 2000).

Whilst the results of this study cannot confirm the potential of ECC RE as a clinical diagnostic tool or as a therapeutic strategy due to differences between the experimental protocol and common exercise practice, it provides novel information on the valuable topic of ventricular ε. This study also provides an insight into cardiovascular changes transiently occurring during brief exposure to an alternative exercise type and in addition, provides comparative data to other studies investigating transient CON and isometric changes. Finally, the results of this study may help work towards a future reference range of ventricular ε values in response to varied exercise modalities.

## Limitations

There are several study limitations that require attention: first, the study sample encompassed only healthy recreationally active males between 18 and 35 years old; therefore, any potential implications for the wider population can only be speculated. In addition, the physiological responses observed during this study can only be described as transient modifications that take place during a single rep at each intensity. Physiological responses to multiple repetitions or training requires further investigation. Additionally, due to the nature of exercise ultrasonography and STI, image quality somewhat varied, through individual differences and torso movement. Although ~ 73% of all images ranked satisfactory or above using the study image quality scale; images of poorer quality existed and may impair analytical accuracy to an extent. Finally, LV ɛ investigations were limited to a single apical view due to the time constraints of imaging during muscular contractions. Therefore, changes may have occurred in other planes or within circumferential and radial ɛ that could not be captured during each recording.

## Conclusion

Results from this study exhibit LV global longitudinal systolic function is transiently unaffected during the performance of brief lower limb ECC RE irrespective of intensity, independent of haemodynamic and chronotropic augmentations. These outcomes may provide foundations for further investigations of the use of large muscle group ECC RE for diagnostic or therapeutic interventions. Future investigations should assess the LV functional responses to long-term ECC resistance training or during multiple repetitions using a larger cohort with potential comparison to clinical populations. The evaluation of LV radial, circumferential and twist mechanics during this unique exercise modality may also be justifiable.

## Abbreviations

ε: Strain
1RM: One repetition maximum
AHA: American heart association
ANOVA: Analysis of variance
ASE: American society of echocardiography
BHS: British hypertension society
BF: Body fat
BP: Blood pressure
CON: Concentric
CV: Coefficient of variation
dBP: Diastolic blood pressure
ECC: Eccentric
ECG: Electrocardiogram
HR: Heart rate
IVSd: Interventricular septal thickness at end-diastole
LV: Left ventricle/ left ventricular
LVDd: Left ventricular end-diastolic diameter
M-mode: Motion mode
MAP: Mean arterial pressure
MAPK: Mitogen-activated protein kinase
MVC: Maximal voluntary contraction
PAR-Q: Physical activity readiness questionnaire
PWd: Posterior wall thickness at end-diastole
RE: Resistance exercise
RPP: Rate pressure product
sBP: Systolic blood pressure
SR: Strain rate
STI: Speckle tracking imaging

## References

[CR1] Alegret J, Beltrán-Debón R, La Gerche A, Franco-Bonafonte L, Rubio-Pérez F, Calvo N, Montero M (2015). Acute effect of static exercise on the cardiovascular system: assessment by cardiovascular magnetic resonance. Eur J Appl Physiol.

[CR158] Beaumont A, Hough J, Sculthorpe N, Richards J (2017). Left ventricular twist mechanics during incremental cycling and knee extension exercise in healthy men. Eur J Appl Physiol.

[CR156] Brown J, Jenkins C, Marwick TH (2009). Use of myocardial strain to assess global left ventricular function: a comparison with cardiac magnetic resonance and 3-dimensional echocardiography. Am Heart J.

[CR3] Burns A, La Gerche A, D'hooge J, MacIsaac A, Prior D (2009). Left ventricular strain and strain rate: characterization of the effect of load in human subjects. Eur J Echocardiogr.

[CR4] Carlson D, Dieberg G, Hess N, Millar P, Smart N (2014). Isometric exercise training for blood pressure management: a systematic review and meta-analysis. Mayo Clinic Proc.

[CR5] Chen T, Tseng W, Huang G, Chen H, Tseng K, Nosaka K (2017). Superior effects of eccentric to concentric knee extensor resistance training on physical fitness, insulin sensitivity and lipid profiles of elderly men. Front Physiol.

[CR154] Colberg SR, Sigal RJ, Fernhall B, Regensteiner JG, Blissmer BJ, Rubin RR, Chasan-Taber L, Albright AL, Braun B (2010). Exercise and type 2 diabetes: the American college of sports medicine and the american diabetes association: joint position statement executive summary. Diabetes Care.

[CR155] Dandel M, Hetzer R (2009). Echocardiographic strain and strain rate imaging — clinical applications. Int J Cardiol.

[CR6] Decloedt A, Verheyen T, Sys S, De Clercq D, van Loon G (2011). Quantification of left ventricular longitudinal strain, strain rate, velocity, and displacement in healthy horses by 2-dimensional speckle tracking. J Vet Internal Med.

[CR7] Farsalinos K, Daraban A, Ünlü S, Thomas J, Badano L, Voigt J (2015). Head-to-head comparison of global longitudinal strain measurements among nine different vendors: the EACVI/ASE Inter-Vendor Comparison Study. J Am Soc Echocardiogr.

[CR8] Farthing J, Chilibeck P (2003). The effects of eccentric and concentric training at different velocities on muscle hypertrophy. Eur J Appl Physiol.

[CR10] Fine N, Crowson C, Lin G, Oh J, Villarraga H, Gabriel S (2014). Evaluation of myocardial function in patients with rheumatoid arthritis using strain imaging by speckle-tracking echocardiography. Ann Rheum Dis.

[CR153] Goto K, Ishii N, Kizuka T, Kraemer RR, Honda Y, Takamatsu K (2009). Hormonal and metabolic responses to slow movement resistance exercise with different durations of concentric and eccentric actions. Eur J Appl Physiol.

[CR11] Hettinger T (2017). Physiology of strength.

[CR150] Hill J, Timmis A (2002). Exercise tolerance testing. BMJ.

[CR12] Hopkins W (2015). Spreadsheets for analysis of validity and reliability. Sport Sci.

[CR13] Isner-Horobeti M, Dufour S, Vautravers P, Geny B, Coudeyre E, Richard R (2013). Eccentric exercise training: modalities, applications and perspectives. Sports Med.

[CR14] Kalam K, Otahal P, Marwick T (2014). Prognostic implications of global LV dysfunction: a systematic review and meta-analysis of global longitudinal strain and ejection fraction. Heart.

[CR15] Kocabay G, Muraru D, Peluso D, Cucchini U, Mihaila S, Padayattil-Jose S, Gentian D, Iliceto S, Vinereanu D, Badano L (2014). Normal left ventricular mechanics by two-dimensional speckle-tracking echocardiography. Reference values in healthy adults. Revista Española de Cardiología.

[CR16] Krishnasamy R, Isbel N, Hawley C, Pascoe E, Burrage M, Leano R, Haluska B, Marwick T, Stanton T (2015). Left ventricular global longitudinal strain (GLS) is a superior predictor of all-cause and cardiovascular mortality when compared to ejection fraction in advanced chronic kidney disease. PLoS ONE.

[CR18] Levy P, Machefsky A, Sanchez A, Patel M, Rogal S, Fowler S, Yaeger L, Hardi A, Holland M, Hamvas A, Singh G (2016). Reference ranges of left ventricular strain measures by two-dimensional speckle-tracking echocardiography in children: a systematic review and meta-analysis. J Am Soc Echocardiogr.

[CR20] Mayo J, Kravitz L (1999). A review of the acute cardiovascular responses to resistance exercise of healthy young and older adults. J Strength Cond Res.

[CR21] Mitchell W, Taivassalo T, Narici M, Franchi M (2017). Eccentric exercise and the critically ill patient. Front Physiol.

[CR22] Moreira H, Nwabuo C, Armstrong A, Kishi S, Gjesdal O, Reis J, Schreiner P, Liu K, Lewis C, Sidney S, Gidding S (2017). Reference ranges and regional patterns of left ventricular strain and strain rate using two-dimensional speckle-tracking echocardiography in a healthy middle-aged black and white population: the CARDIA Study. J Am Soc Echocardiogr.

[CR152] Orlandi S, Bielemann R, Martínez-Mesa J, Barros A, Gigante D, Assunção M (2013). The precision of human body composition measurements using air-displacement plethysmography and dual X-ray absorptiometry. Is there any difference between fasting and non-fasting measurements?. Int J Body Compos Res.

[CR25] Overend T, Versteegh T, Thompson E, Birmingham T, Vandervoort A (2000). Cardiovascular stress associated with concentric and eccentric isokinetic exercise in young and older adults. J Gerontol Seri A.

[CR26] Pickering T, Hall J, Appel L, Falkner B, Graves J, Hill M, Jones D, Kurtz T, Sheps S, Roccella E (2005). Recommendations for blood pressure measurement in humans and experimental animals. Circulation.

[CR27] Potter E, Marwick T (2018). Assessment of left ventricular function by echocardiography: the case for routinely adding global longitudinal strain to ejection fraction. JACC Cardiovasc Imaging.

[CR28] Roig M, O’Brien K, Kirk G, Murray R, McKinnon P, Shadgan B, Reid W (2009). The effects of eccentric versus concentric resistance training on muscle strength and mass in healthy adults: a systematic review with meta-analysis. Br J Sports Med.

[CR29] Rösner A, Bijnens B, Hansen M, How O, Aarsæther E, Müller S, Sutherland G, Myrmel T (2008). Left ventricular size determines tissue Doppler-derived longitudinal strain and strain rate. Eur J Echocardiogr.

[CR33] Smiseth O, Torp H, Opdahl A, Haugaa K, Urheim S (2016). Myocardial strain imaging: how useful is it in clinical decision making?. Eur Heart J.

[CR35] Stefani L, Toncelli L, Di Tante V, Vono M, Cappelli B, Pedrizzetti G, Galanti G (2008). Supernormal functional reserve of apical segments in elite soccer players: an ultrasound speckle tracking handgrip stress study. Cardiovas Ultrasound.

[CR36] Stewart G, Yamada A, Haseler L, Kavanagh J, Chan J, Koerbin G, Wood C, Sabapathy S (2016). Influence of exercise intensity and duration on functional and biochemical perturbations in the human heart. J Physiol.

[CR38] Stöhr E, Stembridge M, Esformes J (2015). In vivo human cardiac shortening and lengthening velocity is region dependent and not coupled with heart rate:‘longitudinal’strain rate markedly underestimates apical contribution. Exp Physiol.

[CR39] Stöhr E, Stembridge M, Shave R, Samuel J, Stone K, Esformes J (2017). Systolic and diastolic LV mechanics during and following resistance exercise. Med Sci Sports Exerc.

[CR40] Vallejo A, Schroeder E, Zheng L, Jensky N, Sattler F (2006). Cardiopulmonary responses to eccentric and concentric resistance exercise in older adults. Age Ageing.

[CR41] Vescovi J, Zimmerman S, Miller W, Hildebrandt L, Hammer R, Fernhall B (2001). Evaluation of the BOD POD for estimating percentage body fat in a heterogeneous group of adult humans. Eur J Appl Physiol.

[CR43] Weiner R, Weyman A, Kim J, Wang T, Picard M, Baggish A (2012). The impact of isometric handgrip testing on left ventricular twist mechanics. J Physiol.

[CR44] Wharton G, Steeds R, Allen J, Phillips H, Jones R, Kanagala P, Lloyd G, Masani N, Mathew T, Oxborough D, Rana B (2015). A minimum dataset for a standard adult transthoracic echocardiogram: a guideline protocol from the British Society of Echocardiography. Echo Res Pract.

[CR45] Williams B, Poulter R, Brown M, Davis M, McInnes G, Potter J, Sever P, Thom S (2004). British Hypertension Society guidelines for hypertension management 2004 (BHS-IV): summary. Br Med J.

[CR46] Yamada A, Luis S, Sathianathan D, Khandheria B, Cafaro J, Hamilton-Craig C, Platts D, Haseler L, Burstow D, Chan J (2014). Reproducibility of regional and global longitudinal strains derived from two-dimensional speckle-tracking and doppler tissue imaging between expert and novice readers during quantitative dobutamine stress echocardiography. J Am Soc Echocardiogr.

[CR47] Yingchoncharoen T, Agarwal S, Popović Z, Marwick T (2013). Normal ranges of left ventricular strain: a meta-analysis. J Am Soc Echocardiogr.

